# Tuning of silicon nitride micro-cavities by controlled nanolayer deposition

**DOI:** 10.1038/s41598-022-19255-9

**Published:** 2022-09-05

**Authors:** Dmitry A. Kalashnikov, Gandhi Alagappan, Ting Hu, Nelson Lim, Victor Leong, Ching Eng Png, Leonid A. Krivitsky

**Affiliations:** 1 Institute of Materials Research and Engineering, Agency for Science, Technology, and Research (A*STAR), 2 Fusionopolis Way, #08-03 Innovis, Singapore, 138634 Singapore; 2 Institute of High Performance Computing, Agency for Science, Technology, and Research (A*STAR), Fusionopolis, 1 Fusionopolis Way, #16-16 Connexis, Singapore, 138632 Singapore; 3 Institute of Microelectronics, Agency for Science, Technology, and Research (A*STAR), 2 Fusionopolis Way, #08-02 Innovis, Singapore, 138634 Singapore

**Keywords:** Optics and photonics, Physics

## Abstract

Integration of single-photon emitters (SPEs) with resonant photonic structures is a promising approach for realizing compact and efficient single-photon sources for quantum communications, computing, and sensing. Efficient interaction between the SPE and the photonic cavity requires that the cavity's resonance matches the SPE’s emission line. Here we demonstrate a new method for tuning silicon nitride (Si_3_N_4_) microring cavities via controlled deposition of the cladding layers. Guided by numerical simulations, we deposit silicon dioxide (SiO_2_) nanolayers onto Si_3_N_4_ ridge structures in steps of 50 nm. We show tuning of the cavity resonance exceeding a free spectral range (FSR) of 3.5 nm without degradation of the quality-factor (Q-factor) of the cavity. We then complement this method with localized laser heating for fine-tuning of the cavity. Finally, we verify that the cladding deposition does not alter the position and spectral properties of nanoparticles placed on the cavity, which suggests that our method can be useful for integrating SPEs with photonic structures.

## Introduction

Integrated quantum photonic devices are critical elements for future quantum networks, quantum computers, and sensors^1–8^. One of the essential elements of such a device is a single-photon emitter (SPE), which is coupled to a high Q-factor cavity. Upon excitation of the SPE, the single photon is emitted in the cavity mode and then routed to an optical network for manipulation and detection^9–12^. Such an interface requires a nearly perfect matching of the emission line of the SPE with the resonance line of the cavity. Given the intrinsic uncertainty in the SPE emission line and the cavity's resonances, their matching requires active tuning of the SPE and/or the cavity. While tuning the emission wavelengths of the SPE is possible, for example, by applying electric fields^13–15^, it is arguably more practical to tune the resonance line of the photonic cavity.

Methods of tuning micro-cavities include thermal and electro-optical tuning, application of mechanical stress, and post-fabrication trimming with functionalization of the surface or pattering of the cladding material^16–27^. Most of these methods allow accurate tuning of the cavity resonances with real-time control. However, they are not free from technical challenges. For example, thermal and electrical tuning require a significant amount of electrical power to be delivered into the chip, especially when broadband tuning across a full free spectral range (FSR) is required^18–20,23–26^. Moreover, in this case, the electrical contacts should be positioned next to the cavities, which complicates the fabrication process and might degrade the cavity performance^18^. Methods based on the deposition of a photochromic film or the functionalization with a laser addressable polyelectrolyte strongly depend on the thickness of the applied material, which is hard to control. These methods may also degrade the optical properties of the cavities due to surface modification^16,17^. Post-fabrication trimming of a cladding material requires precise patterning with electron beam lithography, and the cladding itself prevents efficient coupling to integrated SPEs^27^. Stress-based approaches require sophisticated fabrication and a large device size to tune across the full FSR^21,22^.

Silicon nitride (Si_3_N_4_, or SiN) stands out as a material of choice for many device prototypes due to its CMOS compatibility, broad transparency range, and relatively high refractive index (*n* ~ 2)^28–31^. However, the thermo-optic coefficient of Si_3_N_4_ is approximately one order of magnitude lower than for silicon^32^, which makes it challenging to implement thermal-based tuning strategies described above. Moreover, at cryogenic temperatures required to optimally operate many SPEs, the Si_3_N_4_ thermo-optic coefficient becomes even smaller.

In this work, we propose and realize a method for broadband tuning of Si_3_N_4_ cavities. It is based on depositing a silicon dioxide (SiO_2_) cladding of controlled thickness over Si_3_N_4_ cavities with tens of nanometers step size. We show that within the range of thicknesses of the cladding layer from 200 to 500 nm, we can tune the resonance position within one FSR of 3.5 nm. Although this method can only tune the resonance in relatively coarse steps, it can be complemented with other methods for fine tuning. Nevertheless, this method allows us to first bring the cavity resonances and SPE emission wavelength closer to a rough approximation, before fine-tuning with an another approach. To this end, we demonstrate fine tuning of the cavity by applying laser heat after depositing the SiO_2_ cladding. We also show that SiO_2_ deposition does not change the position and spectral properties of the SPEs (a diamond nanocrystal in our case) that were earlier placed on the ring cavity.

## Theoretical model

**Figure 1:**
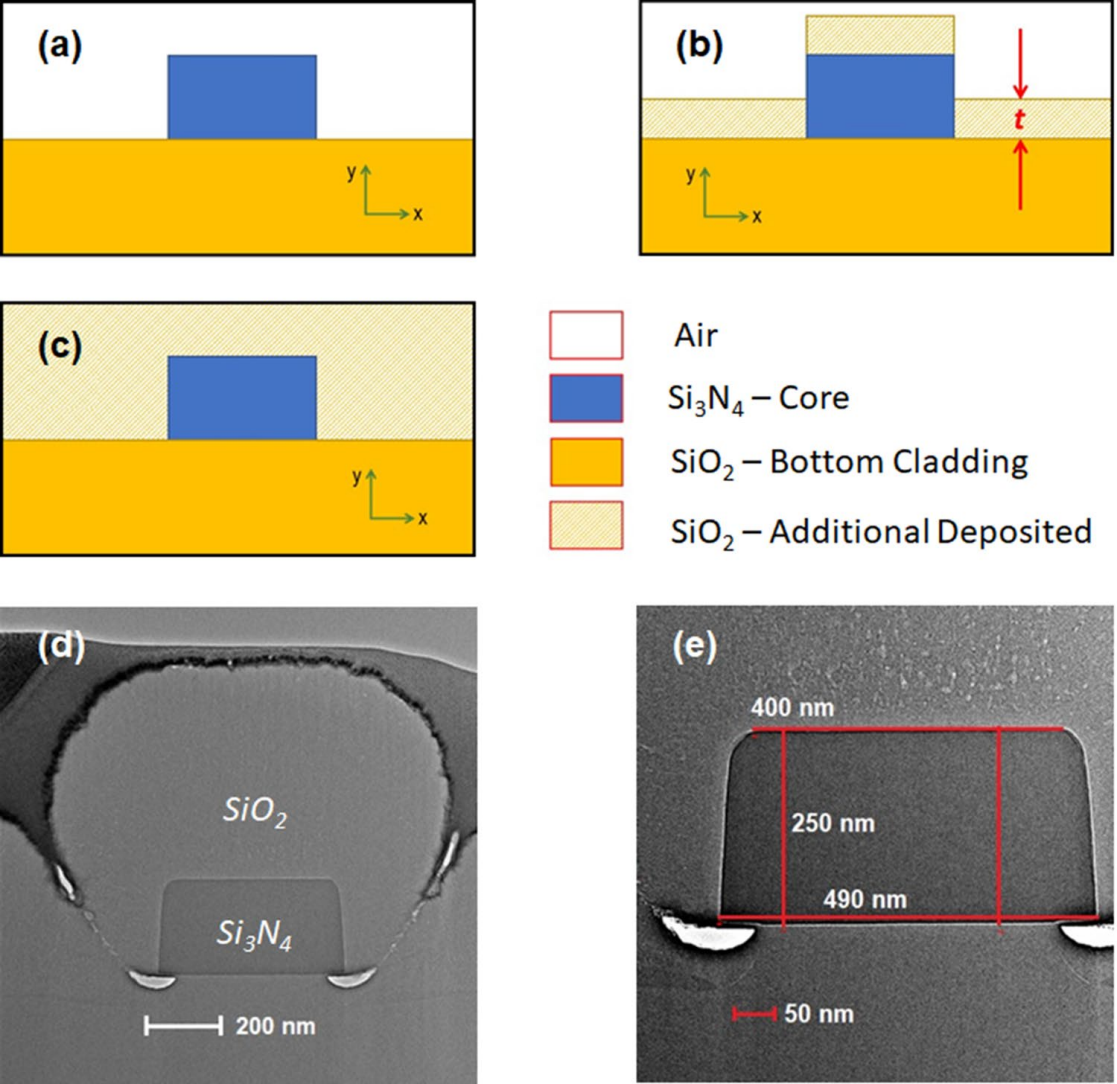
Distribution of SiO_2_ cladding around the Si_3_N_4_ waveguide. (**a**) Schematic of a ridge waveguide with lower and upper cladding being air and SiO_2_, respectively. (**b**) Schematic for theoretical consideration with an additional layer of SiO_2_ deposited. (**c**) A channel waveguide, with the upper and lower cladding being SiO_2_. (**d**) TEM cross-section of a *w* = 480 nm Si_3_N_4_ waveguide after depositing a *t* = 500 nm thick SiO_2_ cladding . (**e**) Measured dimensions of a *w* = 480 nm Si_3_N_4_ waveguide.

We consider a ridge waveguide with the lower and upper cladding being SiO_2_ and air, respectively. Figure 1a shows the cross-section of the ridge waveguide. The Si_3_N_4_ core has a refractive index of 2.087 (measured value at 737 nm) with width *w*, and height *h* = 250 nm. In this work, we consider two nominal design widths, *w* = 330 and 480 nm. The geometrical parameters of the waveguides are designed to support single-mode propagation for both TM and TE polarizations of light with low losses.

We assume that the deposition of SiO_2_ forms a layer with thickness *t* on top of the lower cladding layer and core layer, parallel to the *x*-axis. This is schematically shown in Fig. 1b. The normal line of the additional layer is parallel to *y*-axis. In reality, there will be a thin layer of SiO_2_ (with the normal line along the *x*-axis) surrounding the vertical edges of the core. However, a deterministic value for the thickness of such a layer is hard to obtain. Hence, it has been neglected in the theoretical model. The ridge waveguide (before deposition) represents *t* = 0, and another extreme *t* = ∞ represents a channel waveguide (Fig. 1c). The example of real distribution of SiO_2_ over Si_3_N_4_ waveguide is shown in Fig. 1d,e, (see “Device fabrication” section).

**Figure 2:**
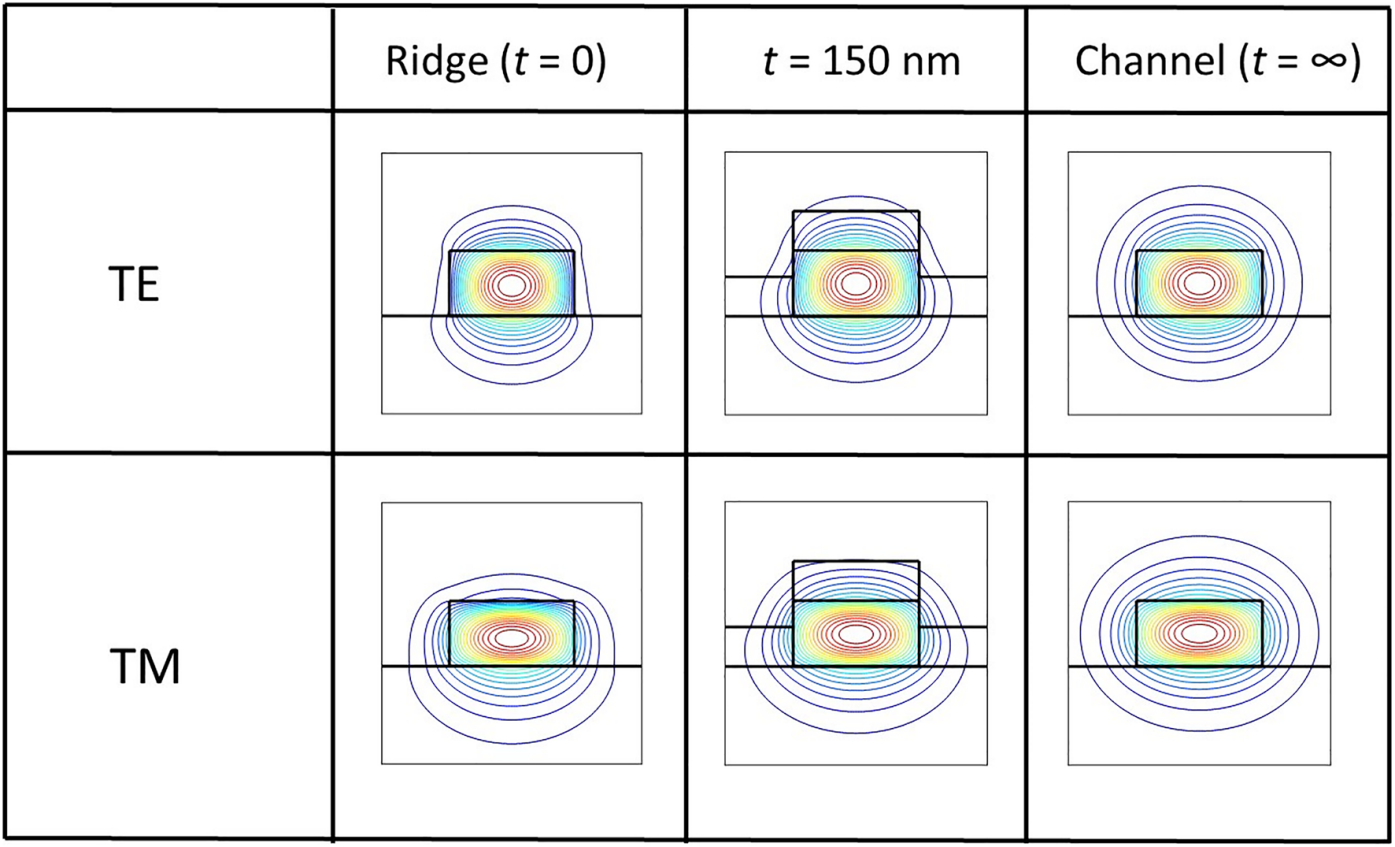
Simulation results for the contour plots of the optical mode (*H*—field) for the waveguide with *h* = 250 nm, *w* = 480 nm. The red (blue) color represents the maxima (minima).

The waveguide optical properties as a function of *t* are obtained by solving the two-dimensional time-independent Maxwell’s equation^33^. In Fig. 2, we exhibit the optical mode profile of the waveguides (*w* = 480 nm) for *t* = 0 (ridge), *t* = 150 nm, and *t* = ∞, for both TE and TM polarizations. For TE polarization, the *E*_*x*_ and *H*_*y*_ components are dominant. On the other hand, for TM polarization, *E*_*y*_ and *H*_*x*_ are dominant. The contrast in refractive index between the upper cladding (air) and the core is larger than the contrast between the lower cladding (SiO_2_) and the core of the ridge waveguide. Therefore, the vertical symmetry is broken in the optical mode pattern. Moreover, the degree of mode penetration is higher in the lower cladding than in the upper cladding. As *t* increases, the upper cladding is modified, and the effective contrast in the refractive index is reduced. Consequently, the asymmetry decreases, and the degree of mode penetration in the upper cladding region increases. In the extreme case of *t* = ∞ (channel waveguide), the symmetry is restored.

The effective refractive index is linked to the group refractive index ${n}_{g}(t)$ by the following equation,

1 $${n}_{\text{eff}}\left(\lambda ,t\right)={n}_{g}\left(t\right)+\lambda F\left(t\right)$$

where $$F(t)=d{n}_{\text{eff}}(t)/d\lambda$$.

If a microring cavity is constructed using a waveguide of effective refractive index ${n}_{\text{eff}}\left(\lambda ,t\right)$, then the resonance frequency can be obtained from the resonance condition $m{\lambda }_{res}={n}_{\text{eff}}\left(\lambda ,t\right)L$, where $L=2\pi R$ is the round–trip length, and *m* is a positive integer^34^. Using this condition, it is straightforward to express ${\lambda }_{res}(t)$ using Eq. (1) as

2 $${\lambda }_{res}\left(t\right)=\frac{\frac{L}{m}{n}_{g}\left(t\right)}{1-\frac{L}{m}F\left(t\right)}$$

**Figure 3:**
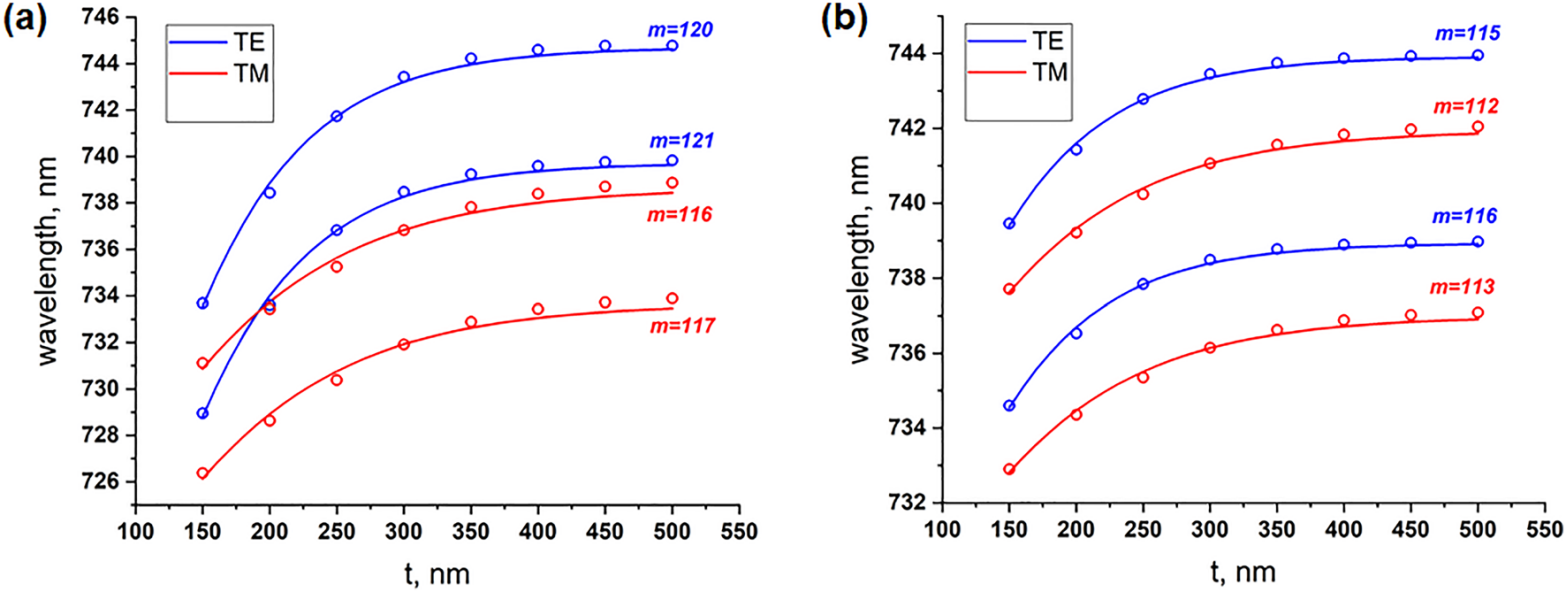
Theoretically estimated resonance wavelengths of the ring cavity (radius *R* = 8 µm) for waveguide widths (**a**) *w* = 330 and (**b**) *w* = 480 nm, respectively. Circles are obtained from Eq. (2), while the solid lines are fits given by Eq. (3).

Figure 3a,b show ${\lambda }_{res}\left(t\right)$ calculated for values of *m* that most closely match the experimental data (see the experimental section below). From these figures we can see that the changes in ${\lambda }_{res}\left(t\right)$ are large and small for small and large *t*, respectively. Therefore, a logistic curve would give a best and representative estimate for the evolution of ${\lambda }_{res}\left(t\right)$. The fitting to a logistic curve can be easily done using a single layer neural network with a sigmoid transfer function, and trained it with data points generated by Eq. (2). The neural network model is employed for a better generalization, and to avoid overfitting^35^. With this formalism, for the range of *t* considered here, we find that ${\lambda }_{res}\left(t\right)$ can be well approximated by the relationship

3 $${\lambda }_{res}\left(t\right)={\lambda }_{\infty }-\frac{B}{1+{e}^{at}}$$

where *B* and *a* are positive constant coefficients. The fitted values of *B* and *a* are tabulated in Table 1. For large *t*, ${\lambda }_{res}\left(t\right)={\lambda }_{\infty }$; for small *t*, using Taylor expansion we have ${\lambda }_{res}\left(t\right)={\lambda }_{\infty }-\frac{B}{2}+\frac{aB}{4}t+\dots$. Clearly, the cavity resonance wavelength varies linearly for small *t*, and eventually saturates as *t* increases. This asymptotic behaviour is true for any mode order *m*.

**Table 1:** The values of *B* and *a* in Eq. (3), obtained from fits to experimental data and theoretical models presented at Fig. 3a,b.

	TE		TM	
	*B*	*a*	*B*	*a*
***w* = 330 nm**				
Expt.	0.20	18.03	0.13	17.72
Theory (mode 1)	0.10	14.08	0.05	10.60
Theory (mode 2)	0.10	14.15	0.05	10.64
***w* = 480 nm**				
Expt.	0.04	16.22	0.05	18.03
Theory (mode 1)	0.04	14.49	0.03	11.41
Theory (mode 2)	0.04	14.55	0.03	11.45

Mode 1 has a higher value of *m* than mode 2.

## Materials and experimental techniques

### Device fabrication

We fabricated Si_3_N_4_ micro-cavity devices consisting of ring cavities (radius *R* = 8 µm) coupled to straight bus waveguides with the same height *h* = 250 nm and width *w* = 330 nm or 480 nm. The gap between the bus and the ring is 160 nm, which is close to the critical gap of the ring cavity that maximizes the bus–cavity coupling. For the efficient coupling of light from a lensed optical fiber, each waveguide has tapered edge couplers (minimum width 150 nm) for the efficient transformation of the TEM_00_ mode of the lensed fiber to propagating modes of the Si_3_N_4_ waveguide.

The fabrication started at a commercial CMOS foundry with a standard 8-in. silicon wafer. A 3.1 µm-thick SiO_2_ layer and a 250 nm-thick Si_3_N_4_ were formed using the thermal oxidation and low-pressure chemical vapor deposition (LPCVD) method. Before deposition of the Si_3_N_4_ layer, chemico-mechanical polishing (CMP) was used to reduce the thickness of the SiO_2_ to 3 µm. Then 248 nm KrF deep ultraviolet (DUV) lithography and inductively coupled plasma (ICP) etch were used to define the Si_3_N_4_ waveguides and ring resonators. This was followed by the upper cladding deposition of 3.4 µm-thick SiO_2_ via plasma-enhanced chemical vapor deposition (PECVD). To expose the Si_3_N_4_ ring resonators to the air, a window was opened in the upper cladding layer of SiO_2_ using lithography and a combination of dry etch (3.1 μm) and wet etch (0.3 μm). The edge coupler for light coupling with fiber was fabricated by the deep etch of the cladding and Si substrate to realize a trench.

The layers of SiO_2_ cladding were deposited in steps of 50 ± 5 nm by ICP-CVD Oxford PlasmaPro System 100 in SiH_4_ and N_2_O atmosphere at 4 mTorr pressure, 150 °C temperature, and 1000 W ICP power. To ensure the thickness accuracy of the cladding layers, each deposition was preceded by calibration measurements, where SiO_2_ was deposited on a blank target and studied under an ellipsometer (J.A. Woollam V-VASE). After each deposition, we measured the cavity resonance wavelengths in our experimental setup. The accuracy of SiO_2_ deposition, its distribution around the waveguide and the cavity were studied via cross-section transmission electron microscope (TEM) images (Fig. 1d,e).

High-pressure high-temperature (HPHT) nanodiamonds containing silicon-vacancy (SiV) centers^36^ were suspended in isopropanol (0.1 wt%), ultrasonicated for 30 min, then randomly deposited on the device by spin-coating at 2000 rpm for 5 min. Under scanning electron microscope (SEM) imaging, we find that approximately 30% of the Si_3_N_4_ cavities had nanodiamonds deposited directly on top of them.

### Measurements

**Figure 4:**
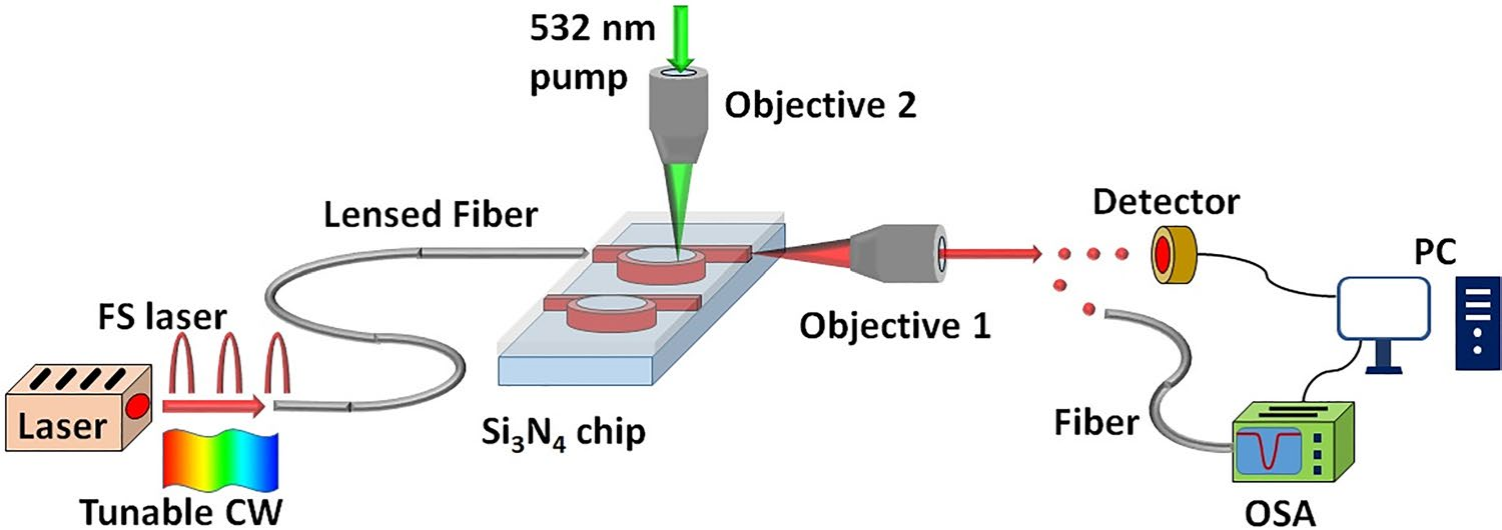
Sketch of the experimental setup: light from broadband fs laser or tunable CW laser is coupled to the lensed fiber. Lensed fiber injects the light into the Si_3_N_4_ micro-cavity device. At the output of the device the transmitted light is collected and collimated with 50 × objective (Objective 1). Then the light is sent either to the photodetector (for tunable CW laser) or to the optical spectral analyzer (OSA) via a single-mode fiber (for fs laser). The 532 nm pump laser is focused onto the micro-cavity device by 100 × objective (Objective 2) for confocal and heating measurements.

We performed measurements at wavelengths of around 737 nm by probing the chip with laser light. It was coupled to the waveguide by a lensed fiber (Oz Optics), and the transmitted light was collected with a 50× objective lens (Olympus) at an opposite edge of the chip, see Fig. 4. We first used attenuated broadband femtosecond (80 fs) laser pulses (Mai Tai, SpectraPhysics) as the input, coupled the transmitted signal to a single-mode fiber, and sent it to an optical spectral analyzer (OSA, Yokogawa AQ6370) with a spectral resolution of 37 pm in the visible range. With its spectral width of over 10 nm, the fs-pulses covered several resonances and allowed a fast estimation of the resonance position. The precise measurement of resonances is then performed by a narrowband (linewidth < 1 MHz) tunable continuous-wave (CW) diode laser (Sacher). We sweep the laser wavelength in the vicinity of the resonance and measure the light intensity passing through the cavity using an optical power meter (Thorlabs) placed after the objective lens. The wavelength of the tunable laser was measured by a wavelength meter (HighFinesse, Model WS-7) with an accuracy of 60 MHz and resolution of 2 MHz. This allowed us to measure the quality factor and fine shifts of the resonance. The devices were placed on a metallic mount which served as an effective heat sink. The ambient temperature fluctuation range was within 0.5 °C over the course of the measurements, within which we did not observe any shift of resonant lines. The experimental data was acquired and post-processed at the PC using self-written software in Python.

A scanning confocal microscope integrated into the test setup was used to measure the fine resonance shift under the laser heating, as well as the spectra of SiV centers in the nanodiamonds before and after SiO_2_ deposition. The 532 nm pump laser was scanned using a fast steering mirror (Newport FSM-300) and projected with a 4f lens system to a 100× objective (NA = 0.9, Nikon), which focused the beam onto the chip. The focused spot size was measured to be 500 nm with a knife blade mounted on a piezo stage (Thorlabs NanoMax Max311). The emission was collected by the same 100× objective, and coupled to a single mode fiber (SMF). The signal from SMF was sent either to an avalanche photodetector (PerkinElmer SPCM-AQRH-14FC) to obtain the confocal scan map, or to a spectrometer equipped with an EMCCD camera (Princeton Instruments, 1200 Grooves/mm).

To apply laser heating experiment, the confocal scan map was used to direct the focused 532 nm laser onto the cavity. The tunable CW laser was swept to measure the shift of the cavity resonance. The position of the beam spot was optimised by moving it across the ring cavity in steps of 400 nm to get the largest resonant line shift.

For the measurements of SiV spectra, the confocal scan map was used to locate nanodiamonds containing SiV on the cavities. Using a 532 nm pump power of 0.4 mW, the SiV photoluminescence was collected via the confocal microscope and sent to the spectrometer, where the collected signal was automatically digitized by the included software (LightField).

## Results and discussions

### Demonstration of tuning using SiO_2_ deposition

**Figure 5:**
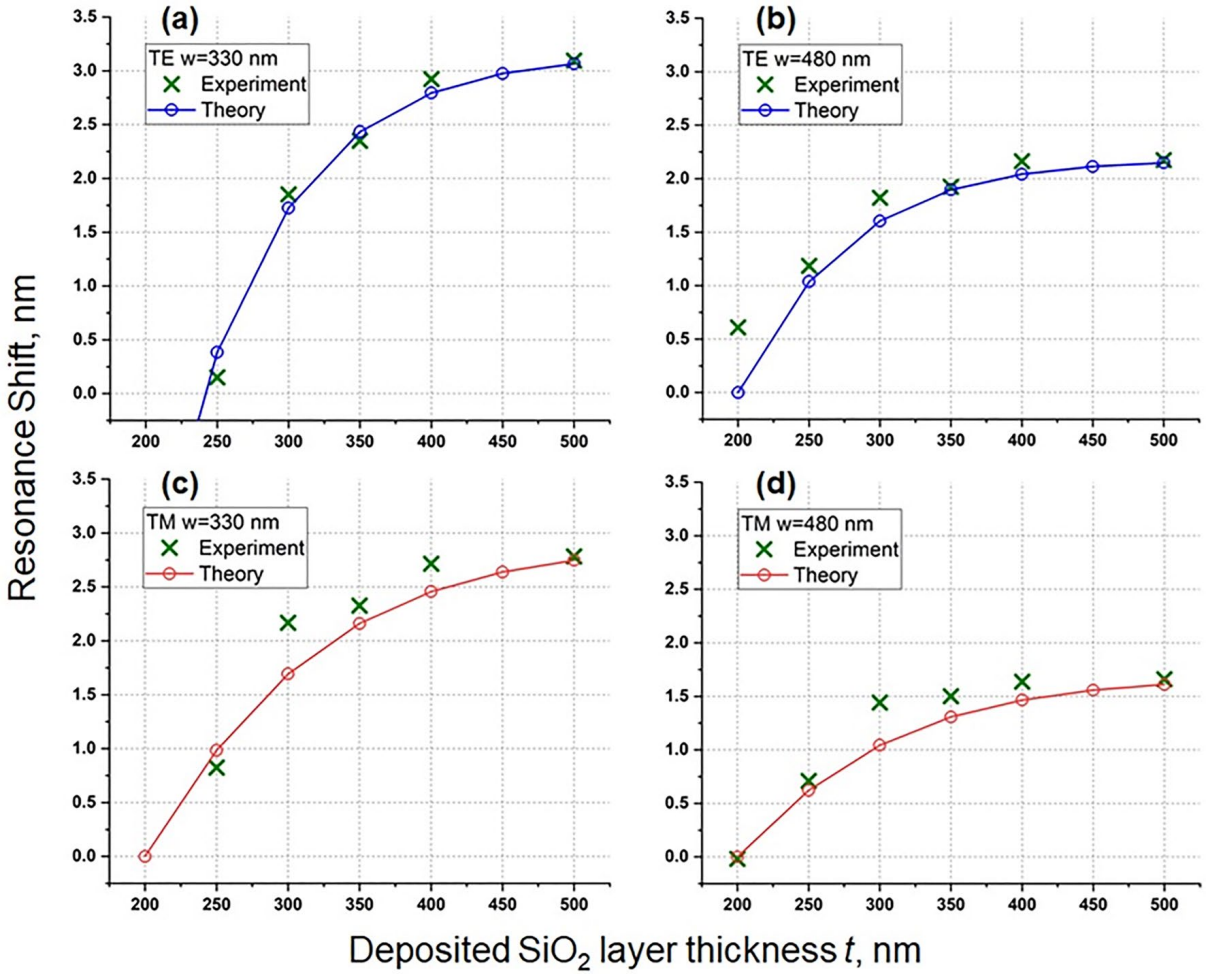
Experimental results for tuning the resonance wavelength as a function of additional SiO_2_ layer thickness. The experimental points are plotted as green crosses, while solid lines represent theoretical curves. Red and blue colors represent TM and TE polarizations, respectively.

For very thin layers deposited onto the cavity, we cannot precisely characterize the resonance shift, as it is larger than one $FSR={\lambda }^{2}/(2\pi {n}_{\text{eff}}R)$. The measured Q-factor is on the order of 10^4^, which we confirm to be unaffected by the deposition of the cladding layers, see Supplementary Fig. S1. Starting from some finite thickness of SiO_2_ layer (200 nm in our case), the resonance shift stays within one FSR, and we can track it within the same order (*m* in Eq. 2). Figure 5 shows the experimentally measured shifts of resonance wavelengths and their theoretical fits as a function of *t*. The theoretical curves are obtained by averaging the numerical derivatives of the respective numerical fits of the two modes in Fig. 3a,b (see Table 1 for the parameters of the fits). We obtain a generally good agreement between the theoretical predictions and experimental measurements; the observed discrepancies are due to uncertainties in material properties as well as some constraints of the theoretical model. For example, it does not include a thin SiO_2_ layer deposited around the vertical edges of the core (with the normal of the layer parallel to *x*-axis, see Fig. 1). Therefore, a good numerical agreement can be obtained by considering the amount of tuning after every increment in the thickness.

A resonance shift of up to 1.5 nm can be obtained when one increases the thickness from 250 to 300 nm (w = 330 nm, TE polarization), while every additional layer of SiO_2_ leads to a decreasing shift in the resonance (see Fig. 5). Devices with a smaller width (w = 330 nm) experience a larger shift as they have optical modes with weaker confinements, and thus are more susceptible to modifications of their refractive index environment.

### Fine-tuning using a laser

While the tuning with SiO_2_ cladding can strongly shift the cavity resonance, the tuning is not very accurate, i.e. it is difficult to exactly match the measured wavelength with the desired theoretically predicted value. We suggest that our method could be complementary to other methods for fine-tuning: depositing the SiO_2_ cladding brings the resonance close to the desired wavelength, and precise tuning can then be achieved by other methods. This two-step approach can be useful when tuning requires significant power consumption and/or heat dissipation. One particular scenario is when cavities with nanoparticles containing SPEs are used at cryogenic temperatures. The heat produced by the chip can degrade the properties of SPEs, for example, by causing the drift or broadening of the emission line.

**Figure 6:**
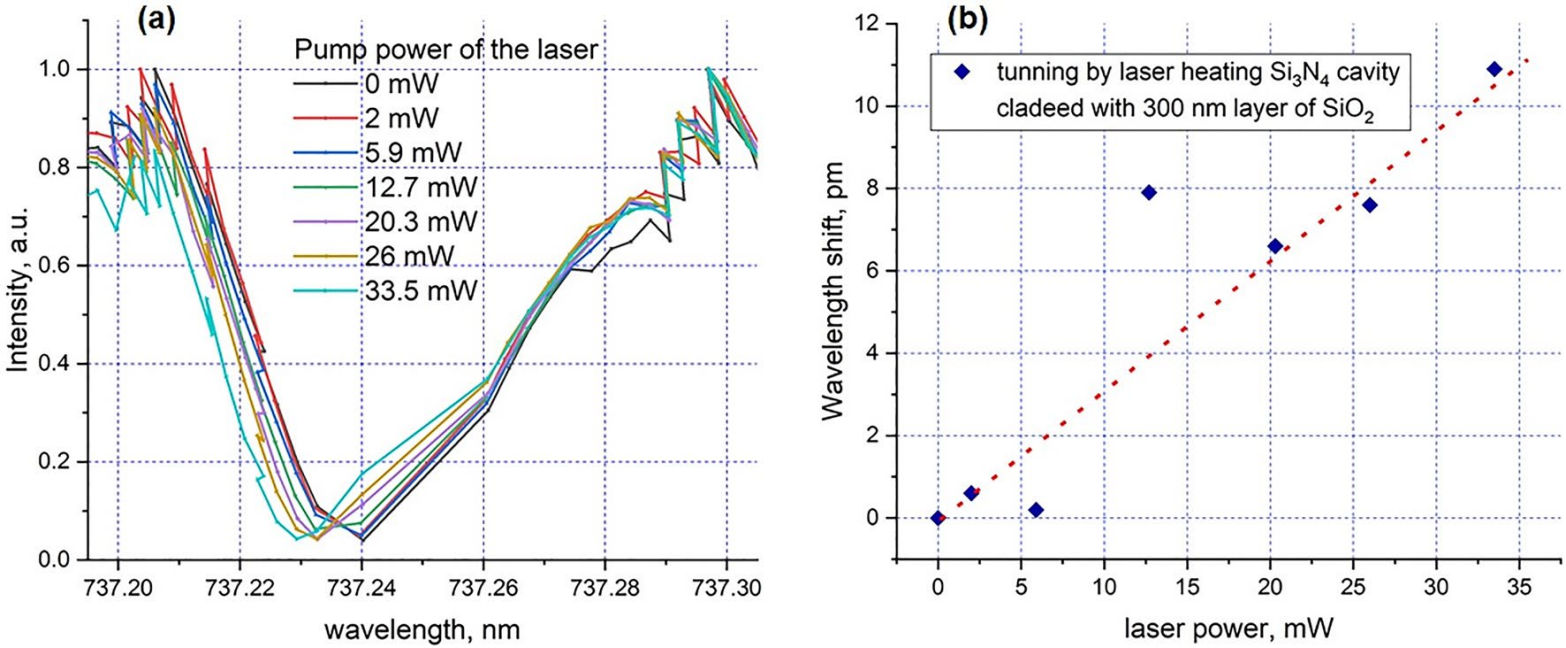
Experimental tuning of the resonance wavelength for Si_3_N_4_ ring cavity clad with 300 nm of SiO_2_ by local heating with 532 nm laser. (**a**) Spectral profiles of the cavity resonances for different laser powers; (**b**) the dependence of reversible shift of the central resonance wavelength on applied pump powers of heating 532 nm laser. The red dotted line is a fit indicating the linear dependence of the refractive index (tuning) on temperature.

We demonstrate this two-step tuning approach by first depositing a 300 nm cladding, then heating the cavity with the 532 nm pump laser in the confocal microscopy setup. By adjusting the laser power, we can precisely tune the resonant frequency line of the cavity within the range of 12 pm, see Fig. 6. We assume that some inaccuracy in the measurements may occur due to the instability of the tunable laser used to measure resonance line profiles, which has limited mode-hop-free tuning range. We verified that the resonance shifts can be attributed to the laser heating by blocking the laser heating beam, which immediately resulted in the return of resonant line to the initial position. We note that the shifts are due to absorption of the heating laser by the SiO_2_ cladding, as we do not observe any shifts with an exposed Si_3_N_4_ cavities_,_ i.e. before SiO_2_ cladding. We note that even though both SiO_2_ and Si_3_N_4_ have approximately the same thermo-optic coefficient, SiO_2_ has an order of magnitude smaller thermal conductivity, which we assume would help to localize the heat at the vicinity of the cavity^37^. To further verify that the laser-induced heating is localised, we moved the laser spot 2 µm away from the cavity. We observed that the cavity resonance line returns to the initial position (without laser heating). Thus we demonstrate that the cavity can be fine-tuned using the combination of cladding deposition and localized laser heating.

We note that we cannot directly determine the heating efficiency; using a simplified assumption that all the laser power is transformed into heat without any losses, we obtain a fine-tuning rate of ~ 0.36 nm/W, i.e. ~ 0.1 FSR per W. Although this is an order of magnitude lower than for cavities with integrated heaters^26,32^, it is sufficient for cavity fine-tuning, and is achievable without the additional fabrication complexity of integrated heaters. However, in practice, SiO_2_ is almost transparent at 532 nm wavelength. To increase the heating efficiency, and hence the tuning rate, we could use UV lasers with a wavelength shorter than 350 nm, where the SiO_2_ absorption is much stronger.

### Integration of the nanoparticles

**Figure 7:**
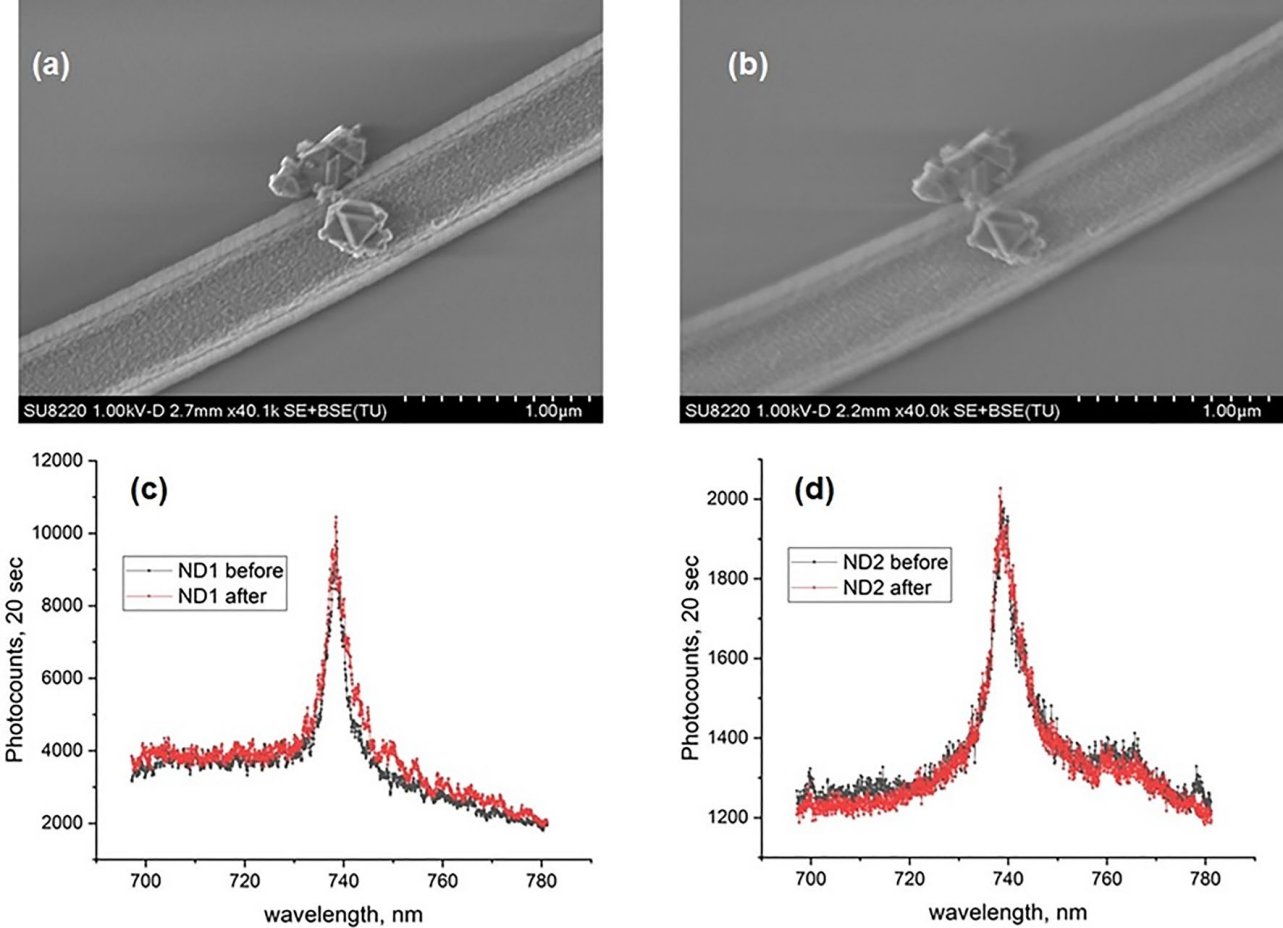
Nanodiamonds before and after SiO_2_ deposition of 500 nm thickness. Position of nanodiamonds placed on top of Si_3_N_4_ ring cavity studied under SEM before (**a**) and after (**b**) deposition by spin-coating. Spectral properties of two different nanodiamonds (**c** and **d**) before (black) and after (red) SiO_2_ deposition obtained in the measuremnts with confocal microscope setup.

Integration of nanoparticles hosting SPEs with photonic platforms is one of the main targets for the successful realization of quantum photonics. We demonstrate that depositing a 500 nm SiO_2_ cladding does not significantly affect the positioning of nanodiamonds deposited by spin-coating on top of the ring cavities. By comparing Scanning Electron Microscope (SEM) images taken before and after cladding, we find that the position of nanodiamonds does not change within the resolution of the SEM, see Fig. 7a,b. We also verified that the SiV emission from the nanodiamonds do not undergo significant changes in brightness or the spectral properties, Fig. 7c,d. This shows that our tuning method is suitable for photonic cavities with integrated nanoparticles.

## Conclusions

We demonstrate a technique for broadband tuning of Si_3_N_4_ microring cavities. The method is based on the deposition of SiO_2_ nanolayers onto the cavities, combined with localized laser heating. Experimental results show good agreement with the theoretical predictions.

The ability to tune the cavity resonance without significant heat dissipation can be important for quantum photonic applications that operate at cryogenic temperatures. In contrast to the application of photochromic or polyelectrolyte films, we do not observe the degradation of the Q-factor. Furthermore, we verify that nanoparticles placed on the cavity are not displaced by the deposition of the cladding, and that their spectral properties do not change.

Our technique can be helpful for small-volume, high Purcell-factor cavities with large FSR, where cavity tuning of up to several nanometers can be required. This will be a valuable addition to the toolkit for developing integrated quantum photonic devices.

## Supplementary Information

Supplementary Figure S1.

## Data Availability

Data may be obtained from the authors upon reasonable request.
