# Supplementary material for: Tuning of silicon nitride micro-cavities by controlled nanolayer deposition
Source: Sci Rep. 2022 Sep 5;12:15074. doi: 10.1038/s41598-022-19255-9 (PMC9445027; doi:10.1038/s41598-022-19255-9)
Supplement: Supplementary file 1 — Supplementary Figure S1. [file 41598_2022_19255_MOESM1_ESM.pdf]

Supplementary Material

Here we compare resonances before and after deposition of an additional layer of SiO_2. As it can be seen from the figures below cladding with SiO_2 does not cause degradation of the Q-factors of resonances in contrast with the application of photochromic or polyelectrolyte films.

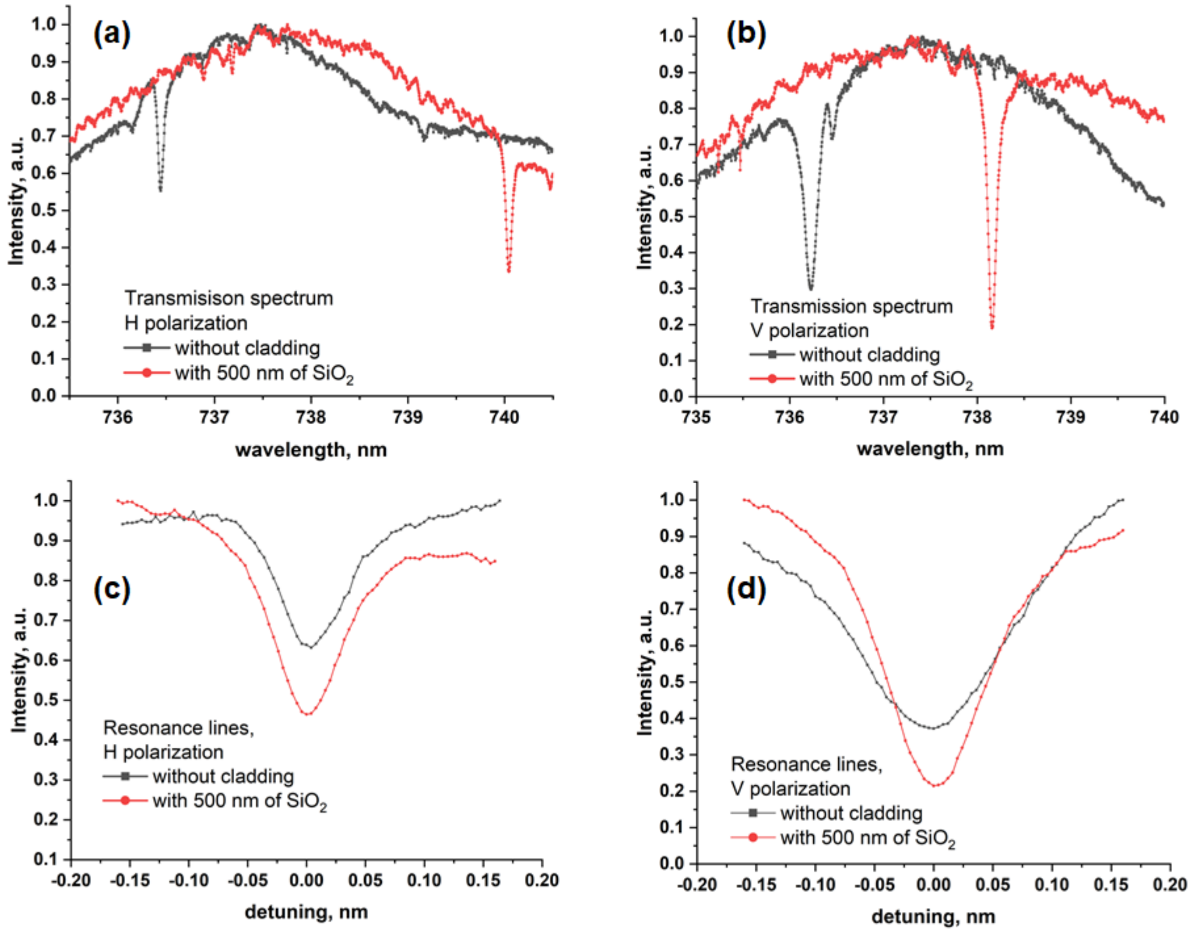

Fig. S1: Comparison of resonance lines of Si_3N_4 ring cavity with R=8 µm and *w*=480 nm before and after SiO_2 cladding. Black denotes results before cladding with SiO_2, red stands for results after deposition of 500 nm of SiO_2. **(a)** and **(b)** Transmission spectrum measured with OSA for Horizontal (TM) **(a)** and Vertical (TE) **(b)** polarizations of the input light. **(c)** and **(d)** One-to-one comparison of resonance lines, **(c)** comparison of resonance lines with Horizontal (TM) polarization from Fig.S1(a), **(d)** comparison of resonance lines with Vertical (TE) polarization from Fig.S1(b)
